# Genes Involved in the PD-L1 Pathway Might Associate with Radiosensitivity of Patients with Gastric Cancer

**DOI:** 10.1155/2020/7314195

**Published:** 2020-09-08

**Authors:** Zixuan Du, Derui Yan, Zhongyang Li, Jincheng Gu, Ye Tian, Jianping Cao, Zaixiang Tang

**Affiliations:** ^1^Department of Biostatistics, School of Public Health, Medical College of Soochow University, Suzhou 215123, China; ^2^Jiangsu Key Laboratory of Preventive and Translational Medicine for Geriatric Diseases, Medical College of Soochow University, Suzhou 215123, China; ^3^School of Radiation Medicine and Protection and Collaborative Innovation Center of Radiation Medicine of Jiangsu Higher Education Institutions, Soochow University, Suzhou 215006, China; ^4^Department of Radiotherapy & Oncology, The Second Affiliated Hospital of Soochow University, Suzhou 215123, China

## Abstract

The PD-1/PD-L1 pathway plays an important role in the treatment of cancers as immune checkpoint. However, the association of genes involved in the PD-L1 pathway and radiosensitivity of gastric cancer has not been fully characterized. This study aims to explore the relationship between the expression levels of genes involved in the PD-L1 pathway and radiosensitivity for gastric cancer patients. A total of 367 patients with clinical survival information and radiotherapy information were obtained in The Cancer Genome Atlas (TCGA). Genes involved in the PD-L1 pathway were categorized into high and low expression level groups according to the median value. The Cox proportional hazards model was used to find the association between gene expression level and radiosensitivity. The results show that high expression levels of CD274, EGFR, RAF1, RPS6KB1, PIK3CA, MTOR, CHUK, NFKB1, TRAF6, FOS, NFATC1, and HIF1A were associated with radiosensitivity of gastric cancer. While low expression level of HRAS was also associated with radiosensitivity in gastric cancer. The rates of a new tumor event and disease progression were lower for radiosensitivity patients than other patients. The relationship between the expression level of CD274 and other genes involved in the PD-L1 pathway is significant. GO (Gene Ontology) analysis shows that the biological process of 13 genes was mainly related to innate immune response activating the cell surface receptor signaling pathway. KEGG analysis demonstrated that 13 genes in gastric cancer are mainly related to the PD-L1 expression and PD-1 checkpoint pathway in cancer. The correlation between the expression level of CD274 and other genes involved in the PD-L1 pathway is significant. The present study offered more evidence for using PD-L1 and genes involved in the PD-L1 pathway as potential biomarkers to predict radiosensitive patients with gastric cancer.

## 1. Introduction

Gastric cancer (GC) is one of the most common and malignant digestive system tumors; it has the second highest incidence and mortality rate of all cancers [1]. According to Chinese cancer statistics, gastric cancer is the second leading cause of cancer death in China and one of the top five malignant tumors [2]. At present, the treatments of gastric cancer mainly include surgical treatment, radiotherapy, chemotherapy, gene therapy, targeted molecular therapy, and comprehensive treatment. Previous studies have reported that radiotherapy is an effective treatment for patients with different stages of gastric cancer [3]. In recent years, radiation therapy in gastric cancer has received increasing attention. However, how to use radiotherapy to improve the patient's quality of life is an urgent problem to be solved.

In the era of precision medicine, searching potential biomarkers and genes to predict radiosensitive patients plays an important role in personalized medicine. One of these examples is the radiosensitivity gene signature, and PD-L1 status of breast cancer patients can be used to select patients [4].

Programmed cell death-ligand encoded by the CD274 gene, also called PD-L1, is the ligand of programmed death-1 (PD-1). PD-L1 is often expressed in T cells, B cells, and other cell types such as DCs, macrophages, mesenchymal stem cells, and a variety of nonhematopoietic cells [5]. The PD-1/PD-L1 pathway activates T cells by delivering costimulatory molecules as the second signal. On the other hand, the PD-1/PD-L1 pathway plays a vital role in maintaining the balance between tolerance and autoimmunity by regulating the degree of activation of T and B cells amongst other immune cell types as critical regulatory immune checkpoints [6]. In solid tumors, the PD-1/PD-L1 inhibitory pathway can be used to suppress the T cell response to promote immune evasion and growth of the tumor by increasing the expression of PD-L1 [7]. PD-L1 expression in the tumor microenvironment has also been studied in multiple solid tumor types including gastric cancer [8], prostate cancer [9], lung cancer [10], and melanoma [11]. In these cancers, PD-L1 overexpression is an indicator of poor prognosis for patient survival.

In this situation, it is essential to understand the regulation mechanism of PD-L1 in cancer. Studies have shown that PD-L1 expression is regulated by transcription factors, signaling pathways, and epigenetic factors [12]. Several signaling pathways include the JAK/STAT pathway, PI3K/Akt signaling pathway, Ras/MEK/ERK pathway, JAK/STAT pathway, HIF-1 signaling pathway, and Toll-like receptor signaling pathway. At the transcriptional level, PD-L1 expression is regulated by several transcriptional factors such as HIF-1, NF*κ*Β, and AP-1. HIF-1 directly binds to the hypoxia-response element in the PD-L1 promoter to regulate PD-L1 expression [13]. Additionally, the late induction of PD-L1 expression of IFN-c is achieved by the regulation of interferon regulatory factor-1 (IRF-1) through Janus-activated kinase (JAK) signal transductor and transcriptional activator (STAT) pathways [14]. Activation of common oncogenic pathways such as the PI3K/Akt signaling pathway and the Ras/MEK/ERK pathway has also been shown to affect tumoral PD-L1 expression. Tristetraprolin (TTP) phosphorylation is caused by Ras activating RAF-MEK-ERK downstream signaling cascade and increased PD-L1 protein expression [15]. The PI3K/Akt pathway is also a key signaling pathway of regulating PL-L1 expression in tumor cells. PD-L1 protein expression may be due to the loss of PTEN in cancer cells [16]. Meanwhile, the Toll-like receptor signaling pathway also regulates the PD-L1 expression.

Most research studies have focused on PD-L1 expression and prognosis, and the relationship between the expression level of PD-L1 and radiosensitivity of gastric cancer patients remains unclear. In addition, genes that regulate PD-L1 expression in cancer might be useful biomarkers for predicting radiosensitive of gastric cancer. Therefore, we investigated the correlation between genes involved in the PD-L1 pathway and radiosensitivity in patients with gastric cancer. For precision medicine, our work offered more evidence for using PD-L1 expression levels and other genes as potential biomarkers to predict radiosensitive for gastric cancer patients.

## 2. Materials and Methods

### 2.1. Data Sources

All gene expression dataset of the gastric cancer patients was downloaded from The Cancer Genome Atlas (TCGA, http://cancergenome.nih.gov/). 443 patients were collected in the TCGA on October 17, 2019. Effective patient survival information was obtained after excluded samples of no survival time or no survival outcome. Clinical data including age, gender, histologic type, pathological stage, and tumor-node-metastasis (TNM) stage were collected from the clinical dataset. Then, the data were obtained by combining clinical data and normalized mRNA sequencing data. Furthermore, the final data were collected with radiotherapy and deleted repeated information. Finally, 367 samples were obtained. The cleaned clinical data are summarized in Table S1. There were 239 men and 128 women in our study. The number of patients who received the radiotherapy was 76, and the number of patients who did not receive radiotherapy was 291.

### 2.2. Analysis Method

**Figure 1:**
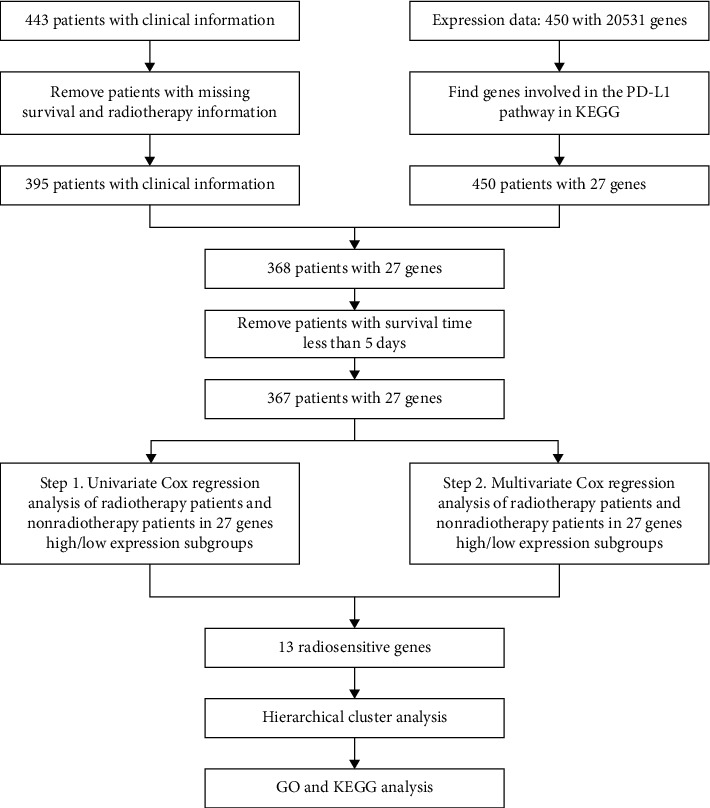
The flow chart of data cleaning and analysis steps.

In the present study, radiosensitive patients were defined as a group of patients who had better overall survival after receiving radiotherapy compared with nonradiotherapy. Radiosensitive gene was defined as the genes which are associated and could be used to identify the radiosensitive patients [17]. The genes involved in the PD-L1 pathway in cancer were found in the Kyoto Encyclopedia of Genes and Genomes (KEGG). A total of 27 genes involved in the present study. Each gene was categorized into high and low groups according to the median value. Kaplan–Meier curves were used to show the survival curves in the high expression group and low expression group. The data clean procedure and radiotherapy sensitive gene selection procedure are shown in Figure 1. The relationship between genes expression levels and radiosensitivity was analyzed by the univariate and multivariate Cox proportional hazards models. The stage divided by the 8^th^ edition of the AJCC Cancer Staging Manual [18]. We also used the Dukes-MAC-like staging system of gastric cancer [19]. The clinical and pathological characteristics of this group were compared by the chi-square test. The functions and pathway of genes were analyzed by GO and KEGG. All statistical analyses were performed by using the *R* packages. In addition, *R* package mice were used to impute the missing values. *p* value 0.05 was considered significant, and all statistical tests were two-sided.

## 3. Results

### 3.1. CD274 and Genes Involved in the PD-L1 Pathway in Cancer Expression Levels

Following the procedure in Figure 1, out of initial 27 genes, 13 genes (CD274, EGFR, HRAS, RAF1, MTOR, RPS6KB1, CHUK, NFKB1, TRAF6, FOS, HIF1A, NFATC1, and PIK3CA) were selected and considered as potential biomarker of radiosensitivity. We investigated each expression level of these genes. As illustrated in Figure S1, the expression levels of 13 genes in gastric cancer were different. The PD-L1 expression level was the lowest among the 13 genes, in which the median expression level was 36.65, and the distribution was mostly concentrated in 19.61–67.74. However, the maximum and minimum values of CD274 expression level were 5728.60 and 1.10. The highest expression level gene of gastric cancer patients was FOS, in which the median expression level was 5007.70, and the maximum and minimum values were 73122.70 and 198.40.

### 3.2. Correlation Analysis of 13 Genes Expression Levels and Clinical Indicators with Survival

In the present study, the Cox proportional hazard model was used to analyze the association between 13 genes expression levels and clinical factors with survival.  Table 1 illustrates the analysis results that univariate analysis and multivariate analysis of clinical indicators and 13 genes expression levels included CD274. The multivariate analysis of each gene expression level is the outcome of gene and clinical factors. The results showed that radiotherapy can improve the patient's overall survival. For clinical indicators, the univariate analysis revealed that T stage (*p*=0.004), M stage (*p*=0.010), N stage (*p*=0.001), pathological stage (*p*=0.001), targeted therapy (*p*=0.022), and chemotherapy (*p*=0.034) were significant factors for overall survival; the multivariate analysis revealed that only N stage (*p*=0.041) was a significant factor for overall survival, and N stage could relate with survival. However, the univariate analysis and multivariate analysis revealed that the relationship between each expression level of 13 genes and overall survival is not significant.

### 3.3. Relationship between Expression Levels of 13 Genes and Clinical Indicators

To identify the relationship between each expression level of 13 genes and clinical factors, we next determined which factors were associated with CD274 and other genes via the chi-square test.

Tables 2 and S3–S13 show that each gene expression level of CD274, EGFR, RAF1, PIK3CA, RPS6KB1, CHUK, TRAF6, FOS, NFKB1, and HRAS has no significant associations with clinical indicators. In addition, the relationship between each gene expression level of NFATC1, HIF1A, MTOR, CHUK, and histologic type was statistically significant, which indicated that the expression levels of these four genes are not identical in different pathological types. RPS6KB1 expression level is associated with gender, and NFATC1 expression level is associated with the T stage. There is a significant relationship between each gene expression level of NFATC1, PIK3CA, and pathological stage.

### 3.4. Relationship between Radiotherapy and Survival in the Two Expression Groups

The study focused on whether the relationship between genes involved in the PD-L1 pathway in cancer and radiosensitivity. The main idea was whether the overall survival of patients with high or low expression level was increased after radiotherapy. For each gene, the gastric cancer patients were categorized into two groups according to their median score and performed survival analysis, respectively. One was a high expression level group and other was a low expression level group. High expression levels of CD274, EGFR, RAF1, RPS6KB1, PIK3CA, MTOR, CHUK, NFKB1, TRAF6, FOS, NFATC1, and HIF1A were sensitive to radiotherapy. However, the low expression level of HRAS was associated with radiosensitivity.

**Figure 2:**
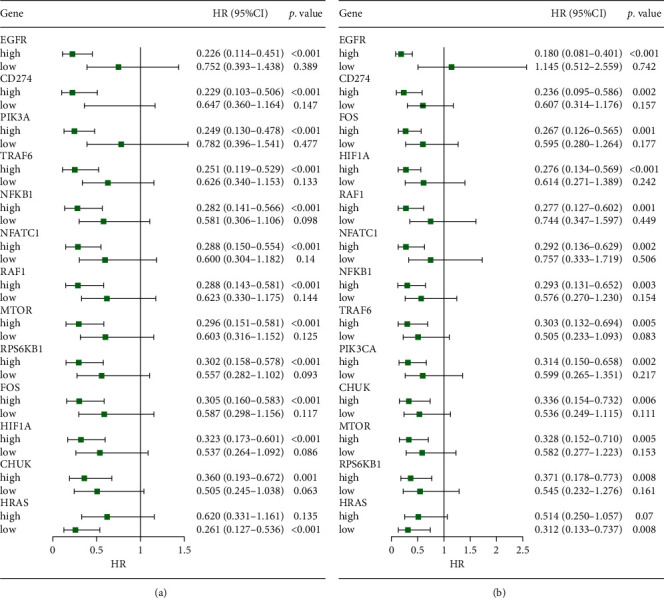
Forest plots for the association analysis between radiotherapy and survival under different expression levels of 13 genes. (a) The forest plot of univariate analysis. (b) The forest plot of multivariable analysis. The adjusted factors include age, gender, histologic type, tumor-node-metastasis (TNM) stage, pathological stage, chemotherapy, and targeted therapy.

Figure 2 demonstrates that the hazard ratio (HR) of each high expression level group of CD274, EGFR, RAF1, PIK3CA, RPS6KB1, MTOR, CHUK, NFKB1, TRAF6, FOS, NFATC1, and HIF1A was still significant after multivariate adjustment was analyzed compared with the low expression level group. For the CD274 gene, the HR and the HR after multivariate adjustment for radiotherapy vs nonradiotherapy were 0.229 (0.103–0.506) and 0.236 (0.095–0.586), respectively. These results indicated that in the high expression level group of these 12 genes, patients who received radiotherapy would have a significant survival benefit and improved their survival rates. For HRAS, the HR after multivariate adjustment was still significant in the low expression level group.

**Figure 3:**
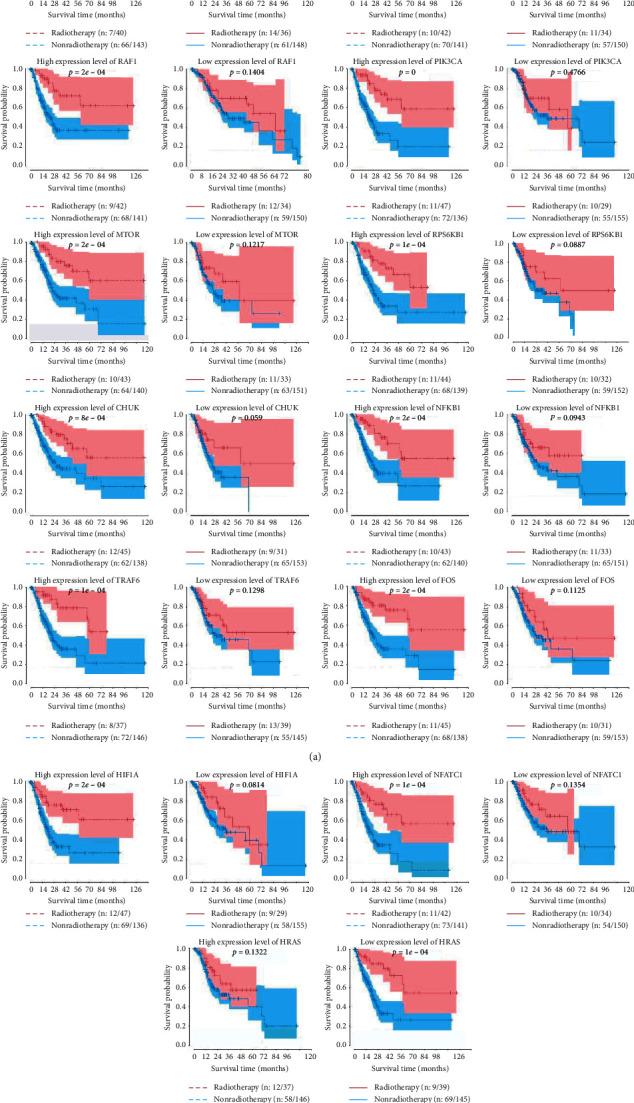
Survival curves under different expression levels of genes involved in the PD-L1 pathway.

The Kaplan–Meier curves for overall survival are graphed as shown in Figure 2. Survival curves of the radiotherapy and nonradiotherapy groups were based on differences in the high and low expression levels of each gene (Figure 3). For CD274, EGFR, RAF1, RPS6KB1, PIK3CA, MTOR, CHUK, NFKB1, TRAF6, FOS, NFATC1, and HIF1A genes, the survival rate of a patient who received radiotherapy in the high expression level group was significantly prolonged compared with patients who did not receive radiotherapy (a). Meanwhile, there was no difference between those who received radiotherapy and those who did not in the low expression group (b). For the HRAS gene, the overall survival rate was significantly higher in the low expression level group than patients who received nonradiotherapy.

### 3.5. Associations among Expressions Levels of 13 Genes and New Tumor Event Rate and Disease Progression Rate

**Figure 4:**
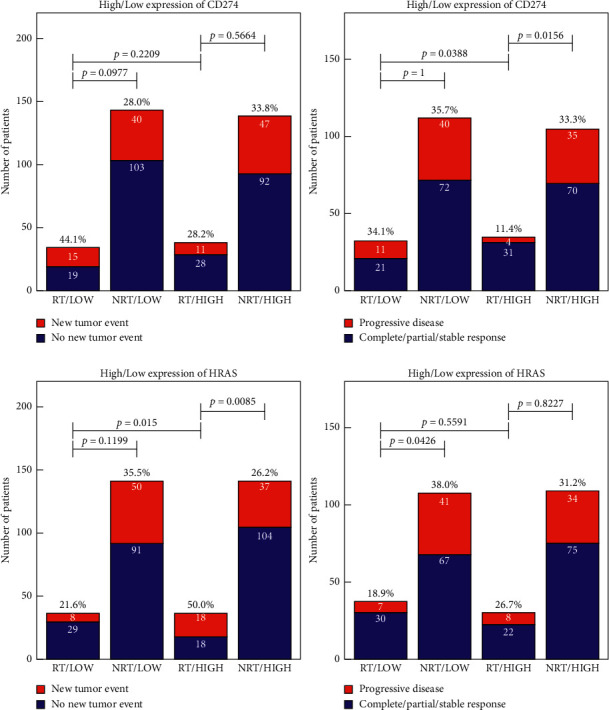
The associations between gene expression level and new tumor event and disease progression rate. The chi-square test was used for comparison of rates of different groups. RT: radiotherapy; NRT: nonradiotherapy; HIGH: high expression level of the gene; LOW: low expression level of gene.

Figures 4 and S2–S12 illustrate the associations among the new tumor event and progressive disease on these two clinical assessment indexes and 13 genes. For new tumor event index and progressive disease rate of CD274, EGFR, RAF1, PIK3CA, MTOR, NFKB1, TRAF6, HIF1A, and NFATC1 genes, the patients in the high expression level group, new tumor event rate, and disease progression rate were not increased and even decreased by received radiotherapy. Meanwhile, the rate of disease progression was also reduced in the highly expressed group after radiotherapy. This result further verified the high expression level of CD274, and these genes associated with radiosensitivity of patients with gastric cancer.

However, for progressive disease rate of RSPS6KS1 gene, under radiotherapy, there was no difference in the new tumor event index between these two expression level group patients. For CHUK and FOS genes, only the rate of disease progression was reduced in the highly expressed group after radiotherapy. For the HRAS gene, in the low expression level group, these two indexes were lower in patients who received radiotherapy compared with the high expression level group.

### 3.6. GO and KEGG Analysis of 13 Genes in Gastric Cancer

**Figure 5:**
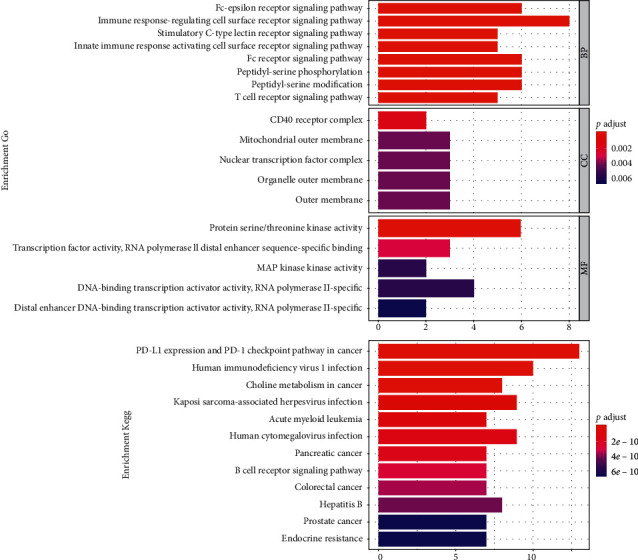
Bar plot for GO and KEGG analysis. BP: biological process; MF: molecular function; CC: cellular component. GO: Gene Ontology; KEGG: Kyoto Encyclopedia of Genes and Genomes.

In the present work, we conducted GO and KEGG analysis of 13 genes in gastric cancer to obtain the biological process, molecular function, cellular component, and pathways. The biological process of 13 genes was mainly related to innate immune response activating the cell surface receptor signaling pathway and the stimulatory C-type lectin receptor signaling pathway (Figure 5). We observed that the molecular functions of 13 genes were mainly involved in protein. Cellular component indicted that 13 genes mainly exit the CD40 receptor and mitochondrial outer membrane. KEGG pathway analysis showed that 13 genes in gastric cancer mainly related to PD-L1 expression and PD-1 checkpoint pathway in cancer and further validated genes from the PD-L1 pathway.

### 3.7. The Correlation between CD274 and Genes Involved in the PD-L1 Pathway in Cancer and Cluster Analysis

**Figure 6:**
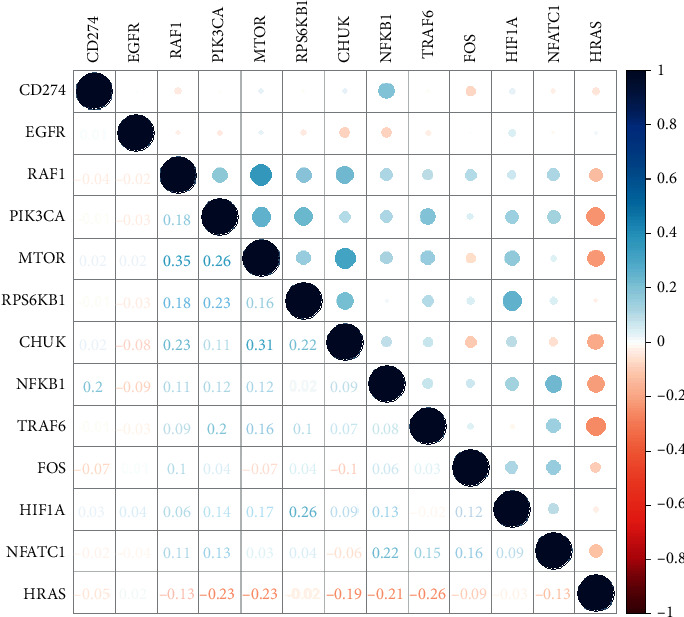
The plot for the correlation of expression levels of genes involved in the PD-L1 pathway.

**Figure 7:**
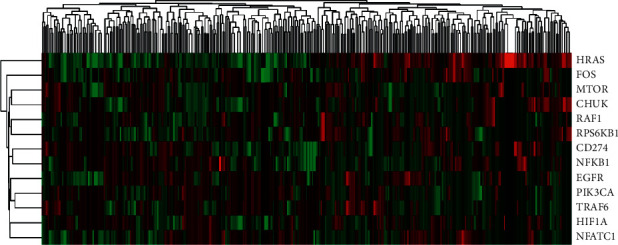
The heatmap of cluster analysis.

**Figure 8:**
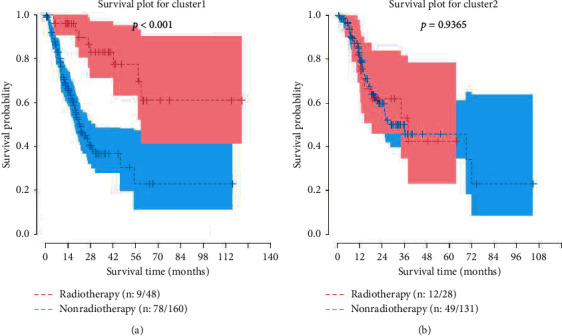
Survival curves under different clusters.

We explored the correlation between CD274 expression level and each expression level of the other 12 genes, and the result is as shown in Figure 6. There are strongly positive correlations in HRAS and HIF1A and FOS and HIF1A. There is a strongly negative correlation in CD274 and CHUK. As shown in Figure 7, all patients are divided into two groups according to the outcome of cluster analysis, cluster 1 includes 195 patients, and cluster 2 includes 172 patients. Furthermore, we plotted the survival plot in these two clusters (Figure 8), for patients in the cluster 1, significantly better survival was observed for patients who received radiotherapy, compared with nonradiotherapy patients.

## 4. Discussion

Radiotherapy is attracting increased attention as a crucial adjuvant therapy for gastric cancer. In past studies, preoperative radiotherapy has progressed in treating gastric cancer, and patients with D1 or D1 plus lymphadenectomy can benefit from postoperative radiotherapy [3]. In the local palliation of gastric cancer, radiation therapy is an effective and well-tolerated modality [20]. However, patients and doctors are concerned about the side effects and long-term effects of radiotherapy. Radiotherapy has more grade adverse events [21]. Therefore, as the development of individualized treatment, finding potential biomarkers, and validating radiation sensitivity, genes in cancer will be of utmost importance for defining its role as a predictive marker and optimizing strategies for cancer radiotherapy.

PD-L1 is encoded by the CD274 gene and binds to the PD-1 receptor expressed on the surface of T cells [5]. Some research studies demonstrated that the CD274 high expression level was associated with a significantly better patient outcome [22], and PD-L1 mRNA levels were upregulated in gastric cancer [8], which are the same as our study. The PD-1/PD-L1 interaction plays a critical role in antigen, and autoimmunity serves as a regulatory checkpoint. A large number of data suggest that, for many cancers, the PD-L1 pathway may be an active immune checkpoint [23]. The PD-1/PD-L1 pathway provided the second signal for effective activation of T cells as costimulatory molecules. Under normal conditions, when the immune system detects cancer cells, the PD-1/PD-L1 pathway can activate T lymphocytes and recognizes tumor cells and kills them. In solid tumors, the silencing of the immune system can be accomplished by increasing the expression of PD-L1 on the surface of tumor cells [7]. Nowadays, the safety and efficacy of anti-PD-1/PD-L1 treatment drugs in melanoma [11], nonsmall cell lung cancer [24], and colorectal carcinoma [25] have been confirmed. The activity and safety of pembrolizumab monotherapy was demonstrated in advanced gastric patients [26].

Studying the relationship between PD-L1 and genes involved in PD-L1 pathways in cancer and radiosensitivity provides important insights into the precision treatment of patients with gastric cancer. In our study, the gastric cancer patients were obtained from TCGA. The median gene score was chosen to categorize the patients into two groups after cleaning the data. One is a high expression group and other is a low expression group. We also used other cutoffs of CD274 such as upper quartile and lower quartile to perform analysis (Figure S13). The results suggested that, when the cutoff is 1/4, the HR of radiotherapy was not significant in these two groups between radiotherapy and nonradiotherapy by multivariate survival analyzed. We also selected 3/4 that larger than 1/2 as other cutoffs, and the HR of radiotherapy in patients with low expression was statistically significant, which may be because of too many higher expression levels patients in the low expression group. These results are consistent with our conclusion that the high expression level of CD274 is related to the radiotherapy sensitivity of gastric cancer.

We divided the patients in the subgroup into patients who received radiotherapy and those who received nonradiotherapy. Then, we performed survival analysis in these two groups and plotted Kaplan–Meier curves for overall survival. Our study found that patients with high CD274 expression level had higher sensitivity, while low expression level suggested that patients were not sensitive to radiotherapy.

Furthermore, we researched genes involved in the PD-L1 pathway which include EGFR, HRAS, RAF1, ALK, PIK3CA, PTEN, AKT3, EGF, MAP2K1, MAPK1, MTOR, RPS6KB1, CHUK, NFKBIA, NFKB1, IFNG, IFNGR1, STAT1, TLR9, TIRAP, MYD88, TRAF6, TICAM1, TICAM2, FOS, HIF1A, and NFATC1 in gastric cancer. Each gene was analyzed by univariate survival and multivariate survival analyzed. Through multivariate analysis, we found that high expression levels of the EGFR, RAF1, RPS6KB1, PIK3CA, CHUK, NFKB1, TRAF6, FOS, NFATC1, and HIF1A genes and the low expression level of the HRAS gene were associated with radiosensitivity. We also studied the relationship between each gene expression level and clinical indicators, and our assumption supported that new tumor event rate and disease progression rate did not significantly increase by receiving radiotherapy.

In recent years, the Dukes-MAC-like staging system was proposed for gastric cancer [19].We also used the Dukes-MAC-like staging system to analyze the relationship between Dukes-MAC-like stage and survival (Table S2). Univariate analysis showed that Dukes-MAC-like stage itself was associated with survival. However, multivariate analysis demonstrated that Dukes-MAC-like stage itself was not associated with survival. Next, we analyzed the associations among expressions levels of 13 genes and Dukes-MAC-like stage. The results showed that each gene expression level of TRAF6, NFATC1, NFKB1, and PIK3A is associated with the Dukes-MAC-like stage.

Shohei Eto et al. demonstrated that disease-free survival (DFS) and overall survival (OS) were significantly poorer in PD-L1-positive patients in gastric cancer [27]. Geng et al. also revealed that the expression levels of PD-L1 are higher and considered as poor prognosis in gastric cancer [28]. However, in this study, there was no significant difference in survival between the high expression group and low expression group.

The genes involved in the PD-L1 pathway in cancer related to radiosensitivity have not been systematically discussed. Preclinical and clinical evidences have demonstrated the activation of antitumor immunity by radiotherapy. The PD-1/PD-L1 pathway plays an important role in immune escape as one of the major mechanisms of cancer. Therefore, the PD-1/PD-L1 pathway after radiotherapy may appropriate systemic antitumor immune activation to improve the curative effect of radiotherapy. In recent years, immunotherapy such as PD-1/PD-L1 immune checkpoint and radiotherapy combined with immunotherapy for gastric cancer have been considered as promising approaches [29]. In cancer treatment, tumor microenvironment is sensitive to treatment with immune checkpoint such as the PD-1/PD-L1 pathway because of radiotherapy. Anti-PD-1/PD-L1 antibodies have potential to relieve immunosuppression caused by radiotherapy in combination therapy [30].

The epidermal growth factor receptor (EGFR) is overexpressed in gastric cancers. The AKT pathway was blocked by the EGFR signaling to suppress the invasion and growth of gastric cancer cells [31]. EGFR expression is associated with the response to radiation. The interaction of EGFR and radiotherapy is complex. Some research studies demonstrated that EGFR inhibitors are related to radiosensitization. EGFR inhibitors may limit tumor repopular through the cytostatic effect [32]. Raf-1 kinase feedback regulation might be associated with radiotherapy sensitivity by enhancing its antiapoptotic function in cancer cells [33]. In glioblastoma, Ras has high activity and sensitivity to radiotherapy [34]. Nuclear factor-kappa B (NF-*κ*B) transcription factors are a key participant in innate and adaptive immune responses as fundamental regulators. The release of NF-*κ*B and activation of the IKK complex were directly involved by the Ras/Raf/MEK/ERK and AKT pathways [35]. The IKK complex enhances the sensitization of ionizing radiation by downregulating either IKK in radiosensitivity [36]. Therefore, Ras/Raf/MEK/ERK and AKT pathways involved CHUK, and RPS6KB1 genes might enhance the sensitizing effect of radiation by downregulation of IKK complex. For RPS6KB1, knockdown of RPS6KB1 increased their sensitivity toward radiation-induced survival inhibition in prostate cancer cells [37]. The activation of the PI3K/AKT pathway involved PI3KCA, and MTOR is linked to radioresistance [38]. Thus, it is supposed that PI3K may overcome radioresistance as a suitable target. Activator protein-1 (AP-1) is encoded by the FOS gene and is related to the control of a variety of cancer cells, such as breast cancer [39] and gastric cancer [2]. The expression level of NFATC1 was decreased in HCC tissues [40] and was significantly upregulated in ovarian cancer [41]. However, more recent findings shed light on the mechanism between FOS, NFATC1, and radiotherapy, which have not been systematically discussed. HIF-1*α* plays an important role in gastric cancer progression and development as a key transcription factor and its overexpression in gastric cancer [42]. Radiotherapy inhibits cervical cancer cell growth through downregulating HOTAIR to inhibit the expression of HIF-1*α* [43]. The reason why various solid tumors are sensitive to radiation is affecting the tumor microenvironment by targeting HIF-1 to reduce the antioxidant capacity of tumors. [44]. We speculated that radiotherapy may control the expression of TRAF6 to influence cell growth and apoptosis.

Only the low expression level of HRAS was associated with radiosensitivity in genes involved in the PD-L1 pathway. HRAS is an attractive target associated with radiation resistance. HRAS gene mutations or EGFR amplification activation can reduce the growth delay of postradiation tumors [45]. Ras signaling to the PI3 kinase-Akt pathway is an important contributor to radiotherapy.

One limitation of our study is that the sample size was small in the TCGA cohort. In this study, there was no external validation study, such as clinical verifications. Then, the study did not consider differences in patient radiation doses. In summary, our study demonstrated the potential predictive and prognostic values of genes involved in the PD-L1 pathway of radiotherapy patients in gastric cancer. These findings provided new insights into the treatment of gastric cancer in the field of radiotherapy, particularly with individualized treatment of cancer patients.

## 5. Conclusion

Genes involved in the PD-L1 pathway might associate with the radiosensitivity of patients with gastric cancer. For precision medicine, our work offered more evidences for using PD-L1 and other genes as potential biomarkers to predict radiosensitive patients for gastric cancer patients.

## Figures and Tables

**Table 1:** Associations of clinical indicators and 13 genes expression levels with total survival.

	Univariate analysis		Multivariate analysis	
	HR (95%CI)	*p* values	HR (95%CI)	*p* values
Radiotherapy				
Yes	0.407 (0.255–0.651)	<0.001	0.417 (0.245–0.710)	0.001
No	1.0000		1.000	
Gender				
Male	1.271 (0.897–1.801)	0.178	1.308 (0.919–1.862)	0.136
Female	1.0000		1.000	
Age				
≥60	1.361 (0.945–1.96)	0.097	1.402 (0.958–2.053)	0.084
<60	1.0000		1.000	
Histologic type				
NOS	1.199 (0.788–1.823)	0.397	1.274 (0.831–1.954)	0.264
DT/MT/SRT	0.903 (0.555–1.47)	0.682	1.112 (0.682–1.840)	0.655
PT/TT	1.0000		1.000	
T Stage				
T3/T4	1.854 (1.215–2.827)	0.004	1.381 (0.855–2.230)	0.187
T1/T2	1.0000		1.000	
M Stage				
M1	1.909 (1.165–3.128)	0.010	1.674 (0.999–2.805)	0.050
M0	1.0000		1.000	
N stage				
N1/N2/N3	1.953 (1.301–2.931)	0.001	1.753 (1.023–3.004)	0.041
N0	1.0000		1.000	
Pathological stage				
III/IV	1.859 (1.309–2.639)	0.001	1.481 (0.897–2.446)	0.125
I/II	1.0000		1.000	
Targeted therapy				
Yes	0.680 (0.489–0.946)	0.022	0.894 (0.430–1.861)	0.765
No	1.0000		1.000	
Chemotherapy				
Yes	0.703 (0.508–0.973)	0.034	0.804 (0.401–1.610)	0.537
No	1.0000		1.000	
CD274				
High	0.813 (0.588–1.123)	0.209	0.734 (0.524–1.027)	0.071
Low	1.0000		1.000	
EGFR				
High	1.098 (0.794–1.518)	0.573	1.209 (0.867–1.686)	0.262
Low	1.0000		1.000	
RAF1				
High	1.046 (0.757–1.445)	0.786	1.249 (0.896–1.741)	0.190
Low	1.0000		1.000	
MTOR				
High	0.906 (0.656–1.252)	0.552	1.008 (0.725–1.401)	0.962
Low	1.0000		1.000	
RPS6KB1				
High	1.154 (0.836–1.595)	0.384	1.283 (0.920–1.789)	0.142
Low	1.0000		1.000	
CHUK				
High	0.865 (0.625–1.197)	0.382	0.941 (0.676–1.309)	0.717
Low	1.0000		1.000	
NFKB1				
High	0.873 (0.632–1.205)	0.409	1.016 (0.729–1.416)	0.926
Low	1.0000		1.000	
TRAF6				
High	1.268 (0.917–1.753)	0.151	1.358 (0.974–1.893)	0.071
Low	1.0000		1.000	
FOS				
High	1.124 (0.813–1.554)	0.478	1.287 (0.923–1.794)	0.137
Low	1.0000		1.000	
HIF1A				
High	1.256 (0.909–1.737)	0.168	1.184 (0.838–1.672)	0.339
Low	1.0000		1.000	
NFATC1				
High	1.291 (0.932–1.787)	0.124	1.364 (0.962–1.934)	0.081
Low	1.0000		1.000	
PIK3CA				
High	1.196 (0.864–1.656)	0.280	1.228 (0.876–1.722)	0.233
Low	1.0000		1.000	
HRAS				
High	0.881 (0.638–1.217)	0.441	0.882 (0.635–1.225)	0.454
Low	1.0000		1.000	

Abbreviations.HR: hazard ratio; NOS: not otherwise specified; DT: diffuse type; MT: mucinous type; SRT: signet ring type; PT: papillary type; TT: tubular type.

**Table 2:** Relationship between expression level and clinical indicators.

	CD274				HRAS			
	High	Low	*χχ*2	*p* Values	High	Low	*χχ*2	*p* Values
Gender			0.135	0.714			1.049	0.306
Female	66	62			69	59		
Male	117	122			114	125		
Age			0.763	0.184			0.034	0.854
<60	50	64			56	58		
≥60	130	120			127	123		
Histologic type			2.061	0.357			2.872	0.238
PT/TT	35	44			35	44		
DT/MT/SRT	53	43			44	52		
NOS	95	94			102	87		
T Stage			2.666	0.103			0.067	0.796
T1/T2	56	42			50	47		
T3/T4	124	141			133	133		
N stage			0.566	0.452			1.473	0.225
N0	59	52			62	50		
N1/N2/N3	122	131			121	132		
M Stage			1.233	0.267			0.000	1.000
M0	171	165			168	168		
M1	12	19			15	16		
Pathological stage			0.104	0.747			3.733	0.053
I/II	88	83			97	74		
III/IV	89	92			83	98		
Dukes-MAC stage			4.992	0.082			5.454	0.065
D	50	45			56	39		
C	71	92			72	91		
A/B	55	41			45	51		

Abbreviations. HR: hazard ratio; NOS: not otherwise specified; DT: diffuse type; MT: mucinous type; SRT: signet ring type; PT: papillary type; TT: tubular type.

## Data Availability

The data used to support this study are available within this article.

## Supplementary Materials

Supplementary Materials Table S1: the information of basic patient characteristics. Table S2: associations of clinical indicators and 13 genes expression levels with total survival. Tables S3–S13: the relationship between expression level and clinical indicators. Figure S1: the box plot for expression levels of genes involved in the PD-L1 pathway. Figures S2–S12: associations among each gene expression level and clinical assessment factors. The chi-square test was used for comparison of rates of different groups. RT: radiotherapy; NRT: nonradiotherapy; HIGH: high expression level of the gene; LOW: low expression level of the gene. Figure S13: the HR values of radiotherapy along with different cutoffs.
