# Supplementary material for: Genes Involved in the PD-L1 Pathway Might Associate with Radiosensitivity of Patients with Gastric Cancer
Source: J Oncol. 2020 Sep 8;2020:7314195. doi: 10.1155/2020/7314195 (PMC7495224; doi:10.1155/2020/7314195)
Supplement: Supplementary Materials — Table S1: the information of basic patient characteristics. Table S2: associations of clinical indicators and 13 genes expression levels with total survival. Tables S3–S13: the relationship between expression level and clinical indicators. Figure S1: the box plot for expression levels of genes involved in the PD-L1 pathway. Figures S2–S12: associations among each gene expression level and clinical assessment factors. The chi-square test was used for comparison of rates of different groups. RT: radiotherapy; NRT: nonradiotherapy; HIGH: high expression level of the gene; LOW: low expression level of the gene. Figure S13: the HR values of radiotherapy along with different cutoffs. [file 7314195.f1.docx]

**Supplementary Materials**

Table S1 Basic patient characteristics

|  | | N | Median(range)/ratio |
| --- | --- | --- | --- |
| Age | 364 | 67.0(30-90) |  |
| NA | 3 |  |  |
| Race |  |  |  |
| Non-White | 100 | 27.24% |  |
| White | 257 | 60.03% |  |
| NA | 10 | 2.72% |  |
| Gender |  |  |  |
| Male | 239 | 65.12% |  |
| Female | 128 | 34.88% |  |
| Status |  |  |  |
| Censor | 219 | 59.67% |  |
| Death | 148 | 40.33% |  |
| Histologic type | |  |  |
| NOS | 189 | 51.50% |  |
| DT/MT/SRT | 96 | 26.16% |  |
| PT/TT | 79 | 21.53% |  |
| NA | 3 | 0.82% |  |
| T stage |  |  |  |
| T3/T4 | 265 | 72.21% |  |
| T1/T2 | 98 | 26.70% |  |
| NA | 4 | 1.09% |  |
| M stage |  |  |  |
| M1 | 31 | 8.40% |  |
| M0 | 336 | 91.55% |  |
| N stage |  |  |  |
| N1/N2/N3 | 253 | 68.94% |  |
| N0 | 112 | 30.52% |  |
| NA | 2 | 0.54% |  |
| Pathological stage | | |  |
| III/IV | 181 | 49.32% |  |
| I/ II | 171 | 46.59% |  |
| NA | 15 | 4.10% |  |
| Dukes-MAC stage |  |  |  |
| D | 95 | 25.96% |  |
| C | 163 | 44.54% |  |
| A/B | 95 | 25.96% |  |
| Targeted therapy | |  |  |
| Yes | 171 | 46.59% |  |
| No | 192 | 52.32% |  |
| NA | 4 | 1.09% |  |
| Chemotherapy | |  |  |
| Yes | 176 | 47.96% |  |
| No | 191 | 52.04% |  |
| Radiotherapy | |  |  |
| Yes | 76 | 20.71% |  |
| No | 291 | 79.29% |  |

Abbreviations：HR: hazard ratio; NOS: not otherwise specified; DT: diffuse type; MT: mucinous type;SRT: signet ring type; PT: papillary type; TT: tubular type.

Table S2: Associations of clinical indicators and 13 genes expression levels with total survival

|  | | Univariate analysis | | Multivariate analysis | |
| --- | --- | --- | --- | --- | --- |
|  | | HR (95%CI) | P values | HR (95%CI) | P values |
| Radiotherapy | |  |  |  |  |
| Yes | | 0.407(0.255-0.651) | <0.001 | 0.413(0.240-0.710) | 0.001 |
| No | | 1.0000 |  | 1.000 |  |
| Gender | |  |  |  |  |
| Male | | 1.271(0.897-1.801) | 0.178 | 1.328(0.930-1.895) | 0.118 |
| Female | | 1.0000 |  | 1.000 |  |
| Age | |  |  |  |  |
| ≥60 | | 1.361(0.945-1.96) | 0.097 | 1.374(0.933-2.024) | 0.108 |
| <60 | | 1.0000 |  | 1.000 |  |
| Histologic type | |  |  |  |  |
| NOS | | 1.199(0.788-1.823) | 0.397 | 1.171(0.706-1.940) | 0.260 |
| DT/MT/SRT | | 0.903(0.555-1.47) | 0.682 | 1.289(0.837-1.986) | 0.250 |
| PT/TT | | 1.0000 |  | 1.000 |  |
| M stage | |  |  |  |  |
| M1 | | 1.909(1.165-3.128) | 0.010 | 1.479(0.760-2.877) | 0.249 |
| M0 | | 1.0000 |  | 1.000 |  |
| N stage | |  |  |  |  |
| N1/N2/N3 | | 1.953(1.301-2.931) | 0.001 | 1.836(1.070-3.150) | 0.027 |
| N0 | | 1.0000 |  | 1.000 |  |
| Pathological stage | |  |  |  |  |
| III/IV | 1.859(1.309-2.639) | | 0.001 | 1.430(0.859-2.381) | 0.169 |
| I/ II | 1.0000 | |  | 1.000 |  |
| Dukes-MAC stage |  | |  |  |  |
| D | 2.283(1.350-71.30) | | 0.024 | 1.335(0.827-2.155) | 0.237 |
| C | 2.198(1.251-64.880) | | 0.029 | 1.246(0.703-2.207) | 0.451 |
| A/B | 1.0000 | |  | 1.000 |  |
| Targeted therapy |  | |  |  |  |
| Yes | 0.680(0.489-0.946) | | 0.022 | 0.856(0.412-1.781) | 0.678 |
| No | 1.0000 | |  | 1.000 |  |
| Chemotherapy |  | |  |  |  |
| Yes | 0.703(0.508-0.973) | | 0.034 | 0.837(0.419-1.672) | 0.615 |
| No | 1.0000 | |  | 1.000 |  |
| CD274 |  | |  |  |  |
| High | 0.813(0.588-1.123) | | 0.209 | 0.733(0.523-1.027) | 0.071 |
| Low | 1.0000 | |  | 1.000 |  |
| EGFR |  | |  |  |  |
| High | 1.098(0.794 -1.518) | | 0.573 | 1.195(0.855-1.668) | 0.297 |
| Low | 1.0000 | |  | 1.000 |  |
| RAF1 |  | |  |  |  |
| High | 1.046(0.757-1.445) | | 0.786 | 1.256(0.900-1.754) | 0.180 |
| Low | 1.0000 | |  | 1.000 |  |
| MTOR |  | |  |  |  |
| High | 0.906(0.656-1.252) | | 0.552 | 1.016(0.727-1.421) | 0.925 |
| Low | 1.0000 | |  | 1.000 |  |
| RPS6KB1 |  | |  |  |  |
| High | 1.154(0.836-1.595) | | 0.384 | 1.287(0.922-1.798) | 0.139 |
| Low | 1.0000 | |  | 1.000 |  |
| CHUK |  | |  |  |  |
| High | 0.865(0.625-1.197) | | 0.382 | 0.929(0.666-1.297) | 0.667 |
| Low | 1.0000 | |  | 1.000 |  |
| NFKB1 |  | |  |  |  |
| High | 0.873(0.632-1.205) | | 0.409 | 1.016(0.724-1.426) | 0.926 |
| Low | 1.0000 | |  | 1.000 |  |
| TRAF6 |  | |  |  |  |
| High | 1.268(0.917-1.753) | | 0.151 | 1.386(0.993-1.936) | 0.055 |
| Low | 1.0000 | |  | 1.000 |  |
| FOS |  | |  |  |  |
| High | 1.124(0.813-1.554) | | 0.478 | 1.284(0.921-1.791) | 0.140 |
| Low | 1.0000 | |  | 1.000 |  |
| HIF1A |  | |  |  |  |
| High | 1.256(0.909-1.737) | | 0.168 | 1.200(0.845-1.704) | 0.309 |
| Low | 1.0000 | |  | 1.000 |  |
| NFATC1 |  | |  |  |  |
| High | 1.291(0.932-1.787) | | 0.124 | 1.352(0.952-1.919) | 0.092 |
| Low | 1.0000 | |  | 1.000 |  |
| PIK3CA |  | |  |  |  |
| High | 1.196(0.864-1.656) | | 0.280 | 1.280(0.908-1.804) | 0.159 |
| Low | 1.0000 | |  | 1.000 |  |
| HRAS |  | |  |  |  |
| High | 0.881(0.638-1.217) | | 0.441 | 0.865(0.621-1.205) | 0.391 |
| Low | 1.0000 | |  | 1.000 |  |

Abbreviations：HR: hazard ratio; NOS: not otherwise specified; DT: diffuse type; MT: mucinous type; SRT: signet ring type; PT: papillary type; TT: tubular type.

Table S3: Relationship between expression level of EGFR and clinical indicators

|  | | EGFR | | | |
| --- | --- | --- | --- | --- | --- |
|  | | High | Low | χ^2^ | P values |
| Gender | |  |  | 0.005 | 0.943 |
| Female | | 65 | 63 |  |  |
| Male | | 120 | 119 |  |  |
| Age | |  |  | 0.002 | 0.966 |
| <60 | | 56 | 58 |  |  |
| ≥60 | | 125 | 125 |  |  |
| Histologic type | | |  | 2.234 | 0.327 |
| PT/TT | 37 | | 42 |  |  |
| DT/MT/SRT | 54 | | 42 |  |  |
| NOS | 90 | | 99 |  |  |
| T stage |  | |  | 0.104 | 0.747 |
| T1/T2 | 47 | | 51 |  |  |
| T3/T4 | 134 | | 131 |  |  |
| N stage |  | |  | 0.973 | 0.324 |
| N0 | 51 | | 61 |  |  |
| N1/N2/N3 | 131 | | 122 |  |  |
| M stage |  | |  | 0.000 | 1.000 |
| M0 | 168 | | 168 |  |  |
| M1 | 15 | | 16 |  |  |
| Pathological stage | | |  | 2.229 | 0.135 |
| I/II | 78 | | 93 |  |  |
| III/IV | 98 | | 83 |  |  |
| Dukes-MAC stage |  | |  | 4.340 | 0.114 |
| D | 49 | | 46 |  |  |
| C | 72 | | 91 |  |  |
| A/B | 55 | | 41 |  |  |

Abbreviations：NOS: not otherwise specified; DT: diffuse type; MT: mucinous type;SRT: signet ring type; PT: papillary type; TT: tubular type

Table S4: Relationship between expression level of RAF1 and clinical indicators

|  | | RAF1 | | | |
| --- | --- | --- | --- | --- | --- |
|  | | High | Low | χ^2^ | P values |
| Gender | |  |  | 1.049 | 0.306 |
| Female | | 69 | 59 |  |  |
| Male | | 114 | 125 |  |  |
| Age | |  |  | 0.499 | 0.480 |
| <60 | | 60 | 54 |  |  |
| ≥60 | | 120 | 130 |  |  |
| Histologic type | | |  | 1.655 | 0.437 |
| PT/TT | 44 | | 35 |  |  |
| DT/MT/SRT | 48 | | 48 |  |  |
| NOS | 89 | | 100 |  |  |
| T stage |  | |  | 2.666 | 0.103 |
| T1/T2 | 56 | | 42 |  |  |
| T3/T4 | 124 | | 141 |  |  |
| N stage |  | |  | 0.141 | 0.707 |
| N0 | 64 | | 58 |  |  |
| N1/N2/N3 | 129 | | 124 |  |  |
| M stage |  | |  | 0.153 | 0.696 |
| M0 | 167 | | 170 |  |  |
| M1 | 17 | | 14 |  |  |
| Pathological stage | | |  | 0.000 | 1.000 |
| I/II | 87 | | 84 |  |  |
| III/IV | 92 | | 89 |  |  |
| Dukes-MAC stage |  | |  | 4.340 | 0.114 |
| D | 49 | | 46 |  |  |
| C | 72 | | 91 |  |  |
| A/B | 55 | | 41 |  |  |

Abbreviations：NOS: not otherwise specified; DT: diffuse type; MT: mucinous type;SRT: signet ring type; PT: papillary type; TT: tubular type

Table S5: Relationship between expression level of MTOR and clinical indicators

|  | | MTOR | | | |
| --- | --- | --- | --- | --- | --- |
|  | | High | Low | χ^2^ | P values |
| Gender | |  |  | 0.084 | 0.772 |
| Female | | 62 | 66 |  |  |
| Male | | 121 | 118 |  |  |
| Age | |  |  | 0.092 | 0.072 |
| <60 | | 55 | 59 |  |  |
| ≥60 | | 126 | 124 |  |  |
| Histologic type | | |  | 6.040 | 0.048 |
| PT/TT | 49 | | 30 |  |  |
| DT/MT/SRT | 44 | | 53 |  |  |
| NOS | 89 | | 99 |  |  |
| T stage |  | |  | 0.938 | 0.333 |
| T1/T2 | 54 | | 44 |  |  |
| T3/T4 | 129 | | 136 |  |  |
| N stage |  | |  | 0.022 | 0.882 |
| N0 | 57 | | 55 |  |  |
| N1/N2/N3 | 125 | | 128 |  |  |
| M stage |  | |  | 0.129 | 0.719 |
| M0 | 169 | | 167 |  |  |
| M1 | 14 | | 17 |  |  |
| Pathological stage | | |  | 0.043 | 0.836 |
| I/II | 85 | | 86 |  |  |
| III/IV | 93 | | 88 |  |  |
| Dukes-MAC stage |  | |  | 4.493 | 0.106 |
| D | 54 | | 41 |  |  |
| C | 73 | | 90 |  |  |
| A/B | 53 | | 43 |  |  |

Abbreviations: NOS: not otherwise specified; DT: diffuse type; MT: mucinous type; SRT: signet ring type; PT: papillary type; TT: tubular type

Table S6: Relationship between expression level of RPS6KB1 and clinical indicators

|  | | RPS6KB1 | | | |
| --- | --- | --- | --- | --- | --- |
|  | | High | Low | χ^2^ | P values |
| Gender | |  |  | 5.468 | 0.019 |
| Female | | 75 | 53 |  |  |
| Male | | 108 | 131 |  |  |
| Age | |  |  | 0.000 | 1.000 |
| <60 | | 57 | 57 |  |  |
| ≥60 | | 124 | 126 |  |  |
| Histologic type | | |  | 1.483 | 0.476 |
| PT/TT | 39 | | 40 |  |  |
| DT/MT/SRT | 53 | | 43 |  |  |
| NOS | 90 | | 99 |  |  |
| T stage |  | |  | 1.950 | 0.163 |
| T1/T2 | 55 | | 43 |  |  |
| T3/T4 | 126 | | 139 |  |  |
| N stage |  | |  | 2.075 | 0.150 |
| N0 | 49 | | 63 |  |  |
| N1/N2/N3 | 134 | | 119 |  |  |
| M stage |  | |  | 0.000 | 0.987 |
| M0 | 167 | | 169 |  |  |
| M1 | 16 | | 15 |  |  |
| Pathological stage | | |  | 0.048 | 0.826 |
| I/II | 83 | | 88 |  |  |
| III/IV | 91 | | 90 |  |  |
| Dukes-MAC stage |  | |  | 2.865 | 0.239 |
| D | 46 | | 49 |  |  |
| C | 90 | | 73 |  |  |
| A/B | 43 | | 53 |  |  |

Abbreviations：NOS: not otherwise specified; DT: diffuse type; MT: mucinous type; SRT: signet ring type; PT: papillary type; TT: tubular type

Table S7: Relationship between expression level of CHUK and clinical indicators

|  | | CHUK | | | |
| --- | --- | --- | --- | --- | --- |
|  | | High | Low | χ^2^ | P values |
| Gender | |  |  | 0.022 | 0.883 |
| Female | | 65 | 63 |  |  |
| Male | | 118 | 121 |  |  |
| Age | |  |  | 0.072 | 0.789 |
| <60 | | 55 | 59 |  |  |
| ≥60 | | 126 | 124 |  |  |
| Histologic type | | |  | 6.040 | 0.049 |
| PT/TT | 49 | | 30 |  |  |
| DT/MT/SRT | 43 | | 53 |  |  |
| NOS | 90 | | 99 |  |  |
| T stage |  | |  | 0.203 | 0.652 |
| T1/T2 | 51 | | 47 |  |  |
| T3/T4 | 129 | | 129 |  |  |
| N stage |  | |  | 0.000 | 0.993 |
| N0 | 55 | | 57 |  |  |
| N1/N2/N3 | 126 | | 127 |  |  |
| M stage |  | |  | 2.208 | 0.137 |
| M0 | 173 | | 164 |  |  |
| M1 | 10 | | 20 |  |  |
| Pathological stage | | |  | 0.571 | 0.450 |
| I/II | 80 | | 91 |  |  |
| III/IV | 93 | | 88 |  |  |
| Dukes-MAC stage |  | |  | 1.161 | 0.560 |
| D | 45 | | 50 |  |  |
| C | 87 | | 76 |  |  |
| A/B | 46 | | 50 |  |  |

Abbreviations: NOS: not otherwise specified; DT: diffuse type; MT: mucinous type; SRT: signet ring type; PT: papillary type; TT: tubular type

Table S8: Relationship between expression level of TRAF6 and clinical indicators

|  | | TRAF6 | | | |
| --- | --- | --- | --- | --- | --- |
|  | | High | Low | χ^2^ | P values |
| Gender | |  |  | 0.005 | 0.943 |
| Female | | 64 | 65 |  |  |
| Male | | 119 | 119 |  |  |
| Age | |  |  | 0.002 | 0.966 |
| <60 | | 56 | 58 |  |  |
| ≥60 | | 125 | 125 |  |  |
| Histologic type | | |  | 2.156 | 0.340 |
| PT/TT | 35 | | 44 |  |  |
| DT/MT/SRT | 53 | | 43 |  |  |
| NOS | 92 | | 97 |  |  |
| T stage |  | |  | 0.975 | 0.324 |
| T1/T2 | 53 | | 45 |  |  |
| T3/T4 | 126 | | 139 |  |  |
| N stage |  | |  | 0.000 | 1.000 |
| N0 | 56 | | 56 |  |  |
| N1/N2/N3 | 127 | | 126 |  |  |
| M stage |  | |  | 0.588 | 0.443 |
| M0 | 165 | | 171 |  |  |
| M1 | 18 | | 13 |  |  |
| Pathological stage | | |  | 0.012 | 0.913 |
| I/II | 84 | | 87 |  |  |
| III/IV | 91 | | 90 |  |  |
| Dukes-MAC stage |  | |  | 8.170 | 0.017 |
| D | 39 | | 56 |  |  |
| C | 95 | | 68 |  |  |
| A/B | 44 | | 52 |  |  |

Abbreviations: NOS: not otherwise specified; DT: diffuse type; MT: mucinous type; SRT: signet ring type; PT: papillary type; TT: tubular type

Table S9: Relationship between expression level of FOS and clinical indicators

|  | | FOS | | | |
| --- | --- | --- | --- | --- | --- |
|  | | High | Low | χ^2^ | P values |
| Gender | |  |  | 0.084 | 0.772 |
| Female | | 62 | 66 |  |  |
| Male | | 121 | 118 |  |  |
| Age | |  |  | 0.000 | 1.000 |
| <60 | | 57 | 57 |  |  |
| ≥60 | | 124 | 126 |  |  |
| Histologic type | | |  | 1.291 | 0.524 |
| PT/TT | 43 | | 36 |  |  |
| DT/MT/SRT | 50 | | 47 |  |  |
| NOS | 88 | | 100 |  |  |
| T stage |  | |  | 0.975 | 0.324 |
| T1/T2 | 52 | | 45 |  |  |
| T3/T4 | 127 | | 139 |  |  |
| N stage |  | |  | 0.093 | 0.760 |
| N0 | 54 | | 58 |  |  |
| N1/N2/N3 | 128 | | 125 |  |  |
| M stage |  | |  | 0.153 | 0.696 |
| M0 | 166 | | 170 |  |  |
| M1 | 17 | | 14 |  |  |
| Pathological stage | | |  | 0.000 | 1.000 |
| I/II | 84 | | 87 |  |  |
| III/IV | 88 | | 93 |  |  |
| Dukes-MAC stage |  | |  | 3.7874 | 0.1505 |
| D | 44 | | 51 |  |  |
| C | 92 | | 71 |  |  |
| A/B | 44 | | 52 |  |  |

Abbreviations: NOS: not otherwise specified; DT: diffuse type; MT: mucinous type; SRT: signet ring type; PT: papillary type; TT: tubular type

Table S10: Relationship between expression level of NFATC1 and clinical indicators

|  | | NFATC1 | | | |
| --- | --- | --- | --- | --- | --- |
|  | | High | Low | χ^2^ | P values |
| Gender | |  |  | 1.545 | 0.214 |
| Female | | 70 | 58 |  |  |
| Male | | 113 | 126 |  |  |
| Age | |  |  | 0.168 | 0.682 |
| <60 | | 59 | 55 |  |  |
| ≥60 | | 122 | 128 |  |  |
| Histologic type | | |  | 16.880 | <0.001 |
| PT/TT | 31 | | 48 |  |  |
| DT/MT/SRT | 65 | | 31 |  |  |
| NOS | 87 | | 102 |  |  |
| T stage |  | |  | 6.883 | 0.009 |
| T1/T2 | 37 | | 61 |  |  |
| T3/T4 | 143 | | 122 |  |  |
| N stage |  | |  | 4.501 | 0.034 |
| N0 | 46 | | 66 |  |  |
| N1/N2/N3 | 136 | | 117 |  |  |
| M stage |  | |  | 0.153 | 0.696 |
| M0 | 166 | | 170 |  |  |
| M1 | 17 | | 14 |  |  |
| Pathological stage | | |  | 7.759 | 0.005 |
| I/II | 70 | | 101 |  |  |
| III/IV | 102 | | 79 |  |  |
| Dukes-MAC stage |  | |  | 12.573 | 0.002 |
| D | 36 | | 59 |  |  |
| C | 82 | | 81 |  |  |
| A/B | 61 | | 35 |  |  |

Abbreviations：NOS: not otherwise specified; DT: diffuse type; MT: mucinous type; SRT: signet ring type; PT: papillary type; TT: tubular type

Table S11: Relationship between expression level of HIF1A and clinical indicators

|  | | HIF1A | | | |
| --- | --- | --- | --- | --- | --- |
|  | | High | Low | χ^2^ | P values |
| Gender | |  |  | 0.589 | 0.443 |
| Female | | 60 | 68 |  |  |
| Male | | 123 | 115 |  |  |
| Age | |  |  | 0.092 | 0.761 |
| <60 | | 55 | 55 |  |  |
| ≥60 | | 126 | 123 |  |  |
| Histologic type | | |  | 7.813 | 0.020 |
| PT/TT | 29 | | 50 |  |  |
| DT/MT/SRT | 55 | | 41 |  |  |
| NOS | 97 | | 91 |  |  |
| T stage |  | |  | 1.159 | 0.282 |
| T1/T2 | 53 | | 44 |  |  |
| T3/T4 | 126 | | 139 |  |  |
| N stage |  | |  | 0.000 | 1.000 |
| N0 | 56 | | 55 |  |  |
| N1/N2/N3 | 127 | | 126 |  |  |
| M stage |  | |  | 0.036 | 0.849 |
| M0 | 167 | | 167 |  |  |
| M1 | 16 | | 14 |  |  |
| Pathological stage | | |  | 2.094 | 0.148 |
| I/II | 77 | | 93 |  |  |
| III/IV | 97 | | 84 |  |  |
| Dukes-MAC stage |  | |  | 1.956 | 0.376 |
| D | 47 | | 48 |  |  |
| C | 89 | | 74 |  |  |
| A/B | 44 | | 52 |  |  |

Abbreviations: NOS: not otherwise specified; DT: diffuse type; MT: mucinous type; SRT: signet ring type; PT: papillary type; TT: tubular type

Table S12: Relationship between expression level of NFKB1 and clinical indicators

|  | | NFKB1 | | | |
| --- | --- | --- | --- | --- | --- |
|  | | High | Low | χ^2^ | P values |
| Gender | |  |  | 0.531 | 0.466 |
| Female | | 60 | 68 |  |  |
| Male | | 123 | 116 |  |  |
| Age | |  |  | 0.065 | 0.799 |
| <60 | | 58 | 56 |  |  |
| ≥60 | | 122 | 128 |  |  |
| Histologic type | | |  | 1.324 | 0.516 |
| PT/TT | 36 | | 43 |  |  |
| DT/MT/SRT | 52 | | 44 |  |  |
| NOS | 93 | | 96 |  |  |
| T stage |  | |  | 1.950 | 0.163 |
| T1/T2 | 55 | | 43 |  |  |
| T3/T4 | 125 | | 140 |  |  |
| N stage |  | |  | 0.006 | 0.937 |
| N0 | 55 | | 57 |  |  |
| N1/N2/N3 | 127 | | 126 |  |  |
| M stage |  | |  | 0.129 | 0.719 |
| M0 | 169 | | 167 |  |  |
| M1 | 14 | | 17 |  |  |
| Pathological stage | | |  | 0.010 | 0.992 |
| I/II | 85 | | 86 |  |  |
| III/IV | 88 | | 93 |  |  |
| Dukes-MAC stage |  | |  | 11.196 | 0.004 |
| D | 38 | | 57 |  |  |
| C | 97 | | 66 |  |  |
| A/B | 42 | | 54 |  |  |

Abbreviations：NOS: not otherwise specified; DT: diffuse type; MT: mucinous type; SRT: signet ring type; PT: papillary type; TT: tubular type

Table S13: Relationship between expression level of PIK3CA and clinical indicators

|  | | PIK3CA | | | |
| --- | --- | --- | --- | --- | --- |
|  | | High | Low | χ^2^ | P values |
| Gender | | 65 | 63 | 0.022 | 0.883 |
| Female | | 118 | 121 |  |  |
| Male | |  |  |  |  |
| Age | |  |  | 1.727 | 0.189 |
| <60 | | 63 | 51 |  |  |
| ≥60 | | 118 | 132 |  |  |
| Histologic type | | |  | 4.414 | 0.110 |
| PT/TT | 31 | | 48 |  |  |
| DT/MT/SRT | 52 | | 44 |  |  |
| NOS | 97 | | 92 |  |  |
| T stage |  | |  | 2.605 | 0.107 |
| T1/T2 | 41 | | 57 |  |  |
| T3/T4 | 138 | | 127 |  |  |
| N stage |  | |  | 4.501 | 0.034 |
| N0 | 46 | | 66 |  |  |
| N1/N2/N3 | 136 | | 117 |  |  |
| M stage |  | |  | 0.000 | 0.987 |
| M0 | 167 | | 169 |  |  |
| M1 | 16 | | 15 |  |  |
| Pathological stage | | |  | 5.058 | 0.025 |
| I/II | 73 | | 98 |  |  |
| III/IV | 100 | | 81 |  |  |
| Dukes-MAC stage |  | |  | 12.647 | 0.002 |
| D | 61 | | 34 |  |  |
| C | 73 | | 90 |  |  |
| A/B | 39 | | 57 |  |  |

Abbreviations: NOS: not otherwise specified; DT: diffuse type; MT: mucinous type; SRT: signet ring type; PT: papillary type; TT: tubular type

**Supplemental figures:**

**Supplemental figure captions**

Figure S1: The box plot for expression levels of genes involved in the PD-L1 pathway.

Figure S2-S12: Associations among each gene expression level and clinical assessment factors. Chi-square test was used for comparison of rates of different groups. RT: radiotherapy; NRT: non-radiotherapy; HIGH: high expression level of the gene; LOW: low expression level of gene.

Figure S13: The HR values of radiotherapy along with different cutoffs.

**Figure S1**

**
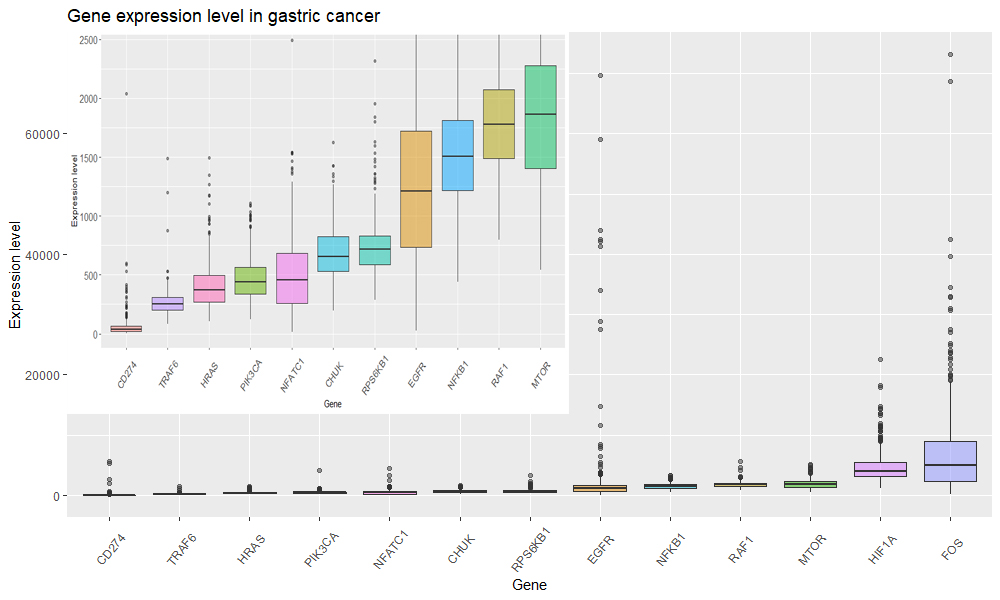
**

Figure S1: The box plot for expression levels of genes involved in the PD-L1 pathway.

**Figure S2-S12**

**
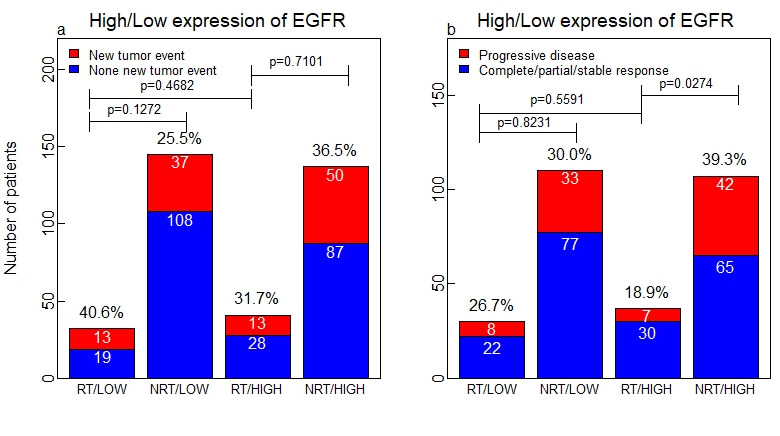
**

**
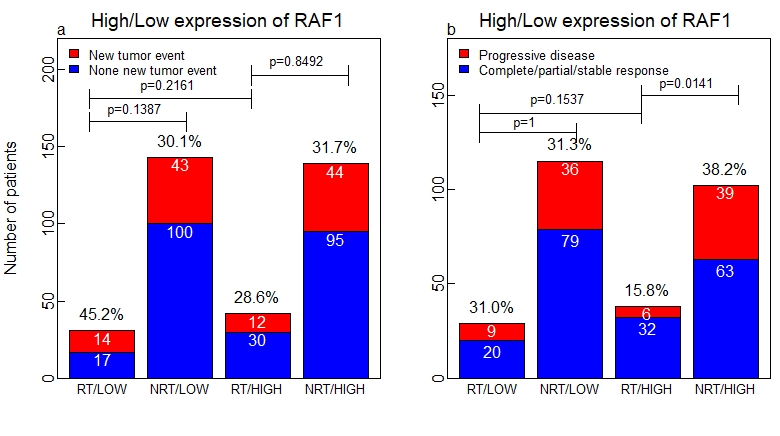
**

**
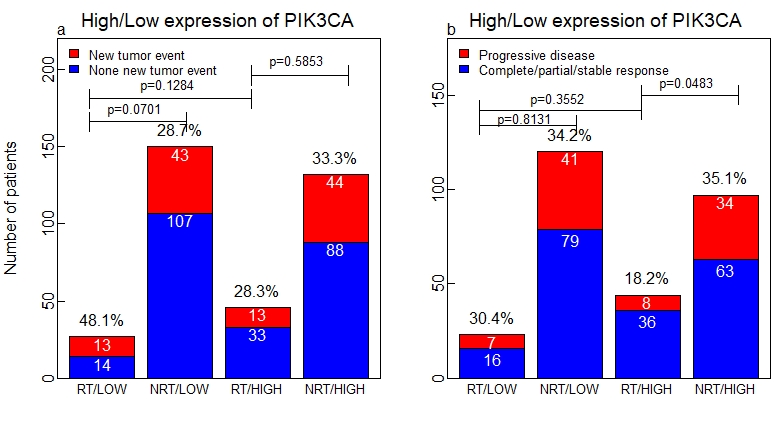
**

**
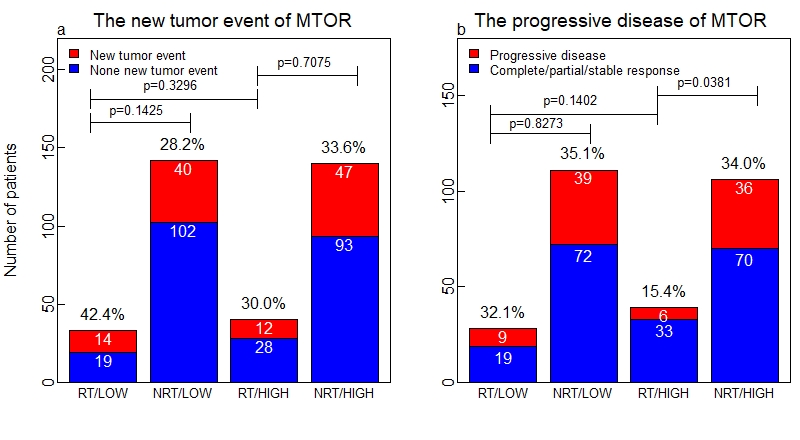
**

**
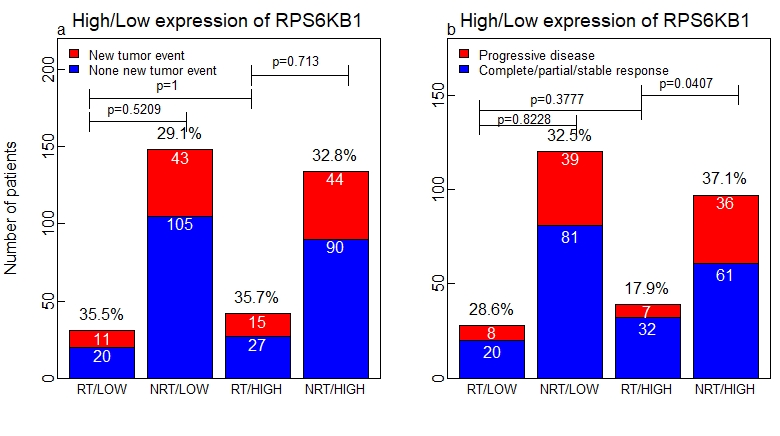
**

**
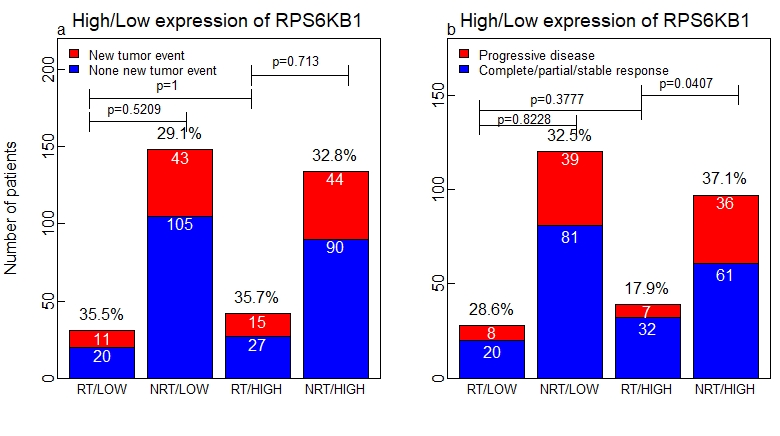
**

**
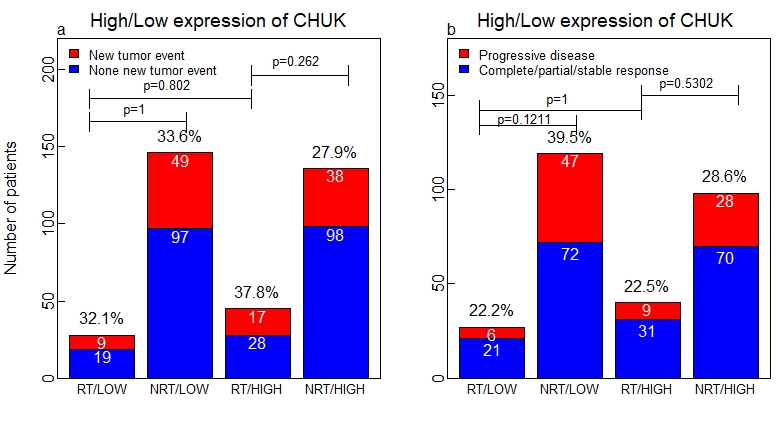
**

**
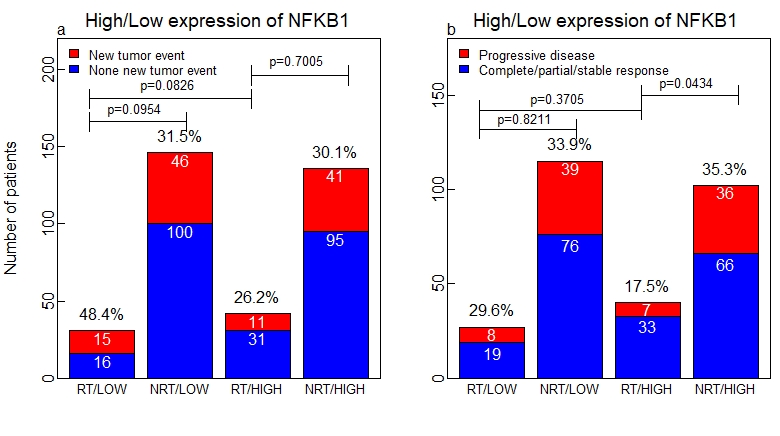
**

**
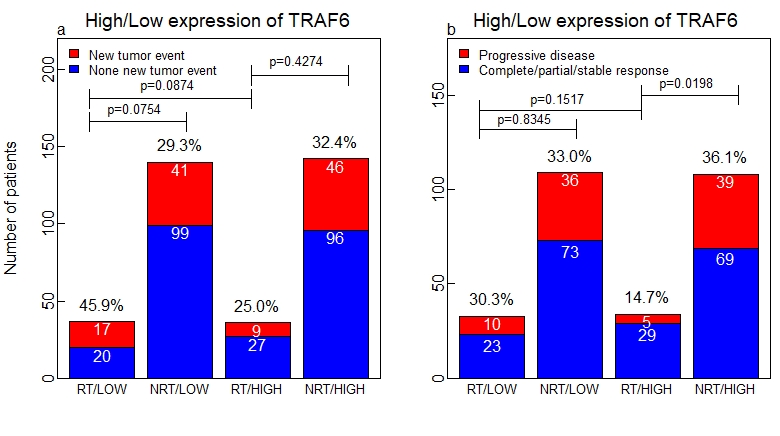
**

**
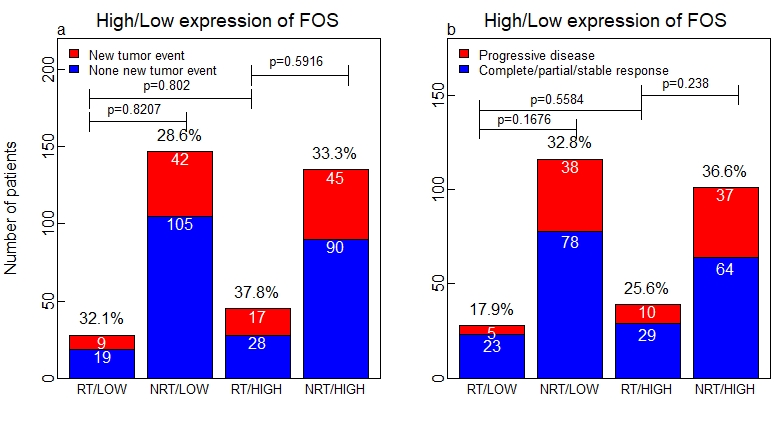
**

**
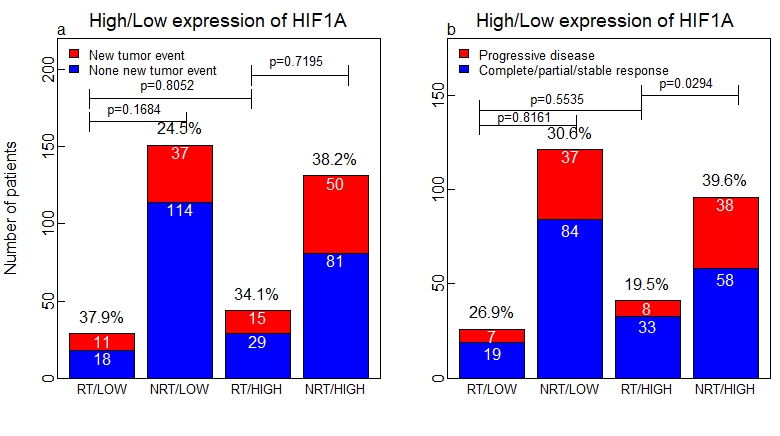
**

**
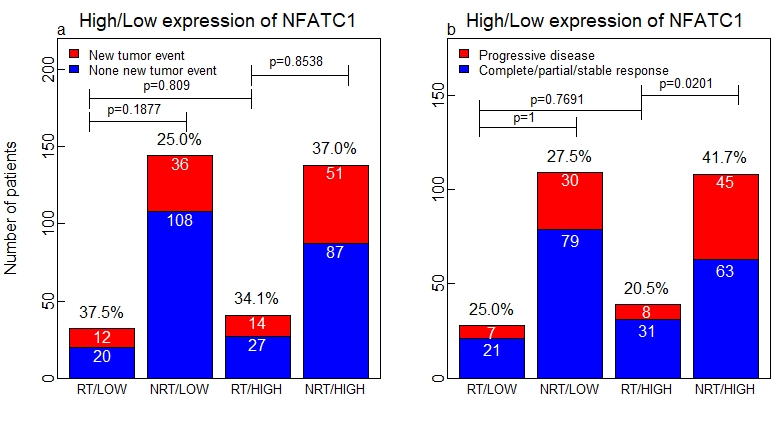
**

Figure S2-S12: Associations among each gene expression level and clinical assessment factors. Chi-square test was used for comparison of rates of different groups. RT: radiotherapy; NRT: non-radiotherapy; HIGH: high expression level of the gene; LOW: low expression level of gene.

**Figure S13**
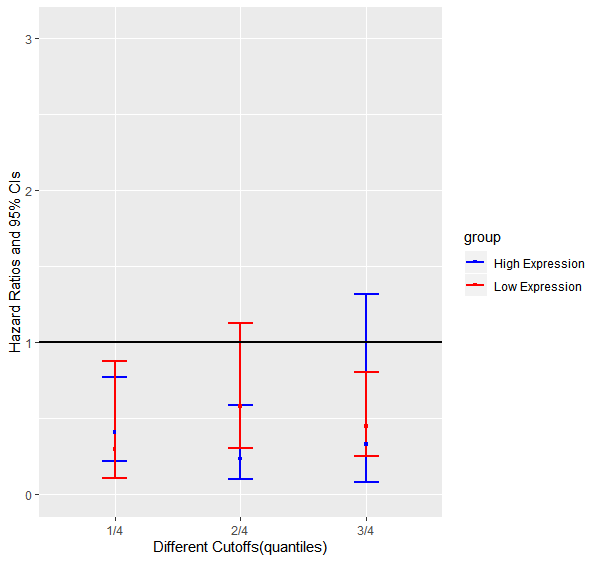


Figure S13: The HR values of radiotherapy along with different cutoffs.
